# Differential Expression and Alternative Splicing Pattern in Female and Male Groups *Pelteobagrus ussuriensis* with Different Growth Rate

**DOI:** 10.3390/ani16030439

**Published:** 2026-01-30

**Authors:** Yanhong Sun, Jian Chen, Pei Li, Lifei Luo, Chuankun Zhu

**Affiliations:** 1 Fisheries Research Institute, Wuhan Academy of Agricultural Sciences, Wuhan 430207, China; chenjian@wuhanagri.com (J.C.); lipei@wuhanagri.com (P.L.); luolifei@wuhanagri.com (L.L.); 2 Jiangsu Key Laboratory for Eco-Agriculture Biotechnology Around Hongze Lake, Jiangsu Collaborative Innovation Center of Regional Modern Agriculture & Environmental Protection, Jiangsu Engineering Laboratory for Breeding of Special Aquatic Organisms, Huaiyin Normal University, Huai’an 223300, China

**Keywords:** full-length RNA sequencing, sex-specific, alternative splicing, growth, differentially expressed genes

## Abstract

Growth rate is a key trait influencing productivity in aquaculture species, and its regulation often differs between males and females. In this study, Nanopore full-length RNA sequencing was used to investigate sex-specific growth regulation in the liver and brain of *Pelteobagrus ussuriensis*. Male and female groups each included three fast-growing and three slow-growing individuals. In liver tissue, 332 differentially expressed genes were identified in males and 266 in females. Male-biased genes were mainly involved in lipid and cholesterol metabolism, including the peroxisome proliferator-activated receptor signaling pathway, whereas females showed broader metabolic regulation involving carbohydrate, amino acid, and lipid metabolism, as well as growth-related genes such as IGFBP1, ESR1, and PGR. In brain tissue, fewer growth-associated differences were observed, with 26 differentially expressed genes in males and 45 in females. Alternative splicing analysis revealed strong tissue specificity, with approximately 2903 events in liver and 7412 in brain, dominated by exon skipping in liver and alternative first exon usage in brain. Isoform-level analysis further identified transcript differences not detected at the gene level, highlighting the importance of transcript diversity in growth regulation.

## Simple Summary

Growth rate is an important factor that affects how efficiently farmed fish produce food; but, males and females may grow differently because they are regulated by different biological processes. In this study, we investigated why some male and female *Pelteobagrus ussuriensis* grow faster than others by comparing fast-growing and slow-growing individuals. We focused on two key organs, the liver and the brain, which play important roles in metabolism and growth control. We found that males and females use different biological pathways to regulate growth. In males, growth differences were mainly related to fat and cholesterol processing, while in females, growth differences involved a wider range of processes, including the use of sugars, fats, and proteins. Some genes directly related to growth and hormone regulation were also different between fast- and slow-growing fish. In the brain, growth-related differences were much smaller but still showed clear sex-specific patterns. We also discovered that fish produced different forms of the same gene, which influenced growth even when overall gene activity appears unchanged. These findings improve our understanding of how growth is regulated and may help develop better strategies for fish breeding and sustainable food production.

## 1. Introduction

Sexual size dimorphism (SSD) is a well documented phenomenon in numerous animal taxa, including many species of economic importance in aquaculture. SSD refers to the difference in body size between males and females of the same species, with one sex generally achieving a larger body size than the other [1]. This trait is often influenced by selective pressures such as reproductive strategies, growth rates, and environmental adaptations [2]. In aquaculture species, SSD can significantly affect productivity, as larger individuals tend to have higher market value, enhanced growth efficiency, and different nutritional requirements. For instance, in many fish species such as Nile tilapia (*Oreochromis niloticus*) and rainbow trout (*Oncorhynchus mykiss*), females tend to reach larger sizes than males under similar rearing conditions [3]. However, there are also notable examples of male-biased size dimorphism, such as in the half-smooth tongue sole (*Cynoglossus semilaevis*) [4] and Ussuri catfish (*Pelteobagrus ussuriensis*) [5], where males are larger than females, as well as in other species like the European eel (*Anguilla anguilla*) and certain species of shrimp and crab [6].

Understanding the underlying mechanisms of SSD in aquaculture species is crucial for optimizing breeding programs, as sex-specific growth traits can inform selective breeding strategies aimed at maximizing yield. Recent advances in molecular genetics, particularly RNA sequencing (RNA-Seq) and full-length RNA sequencing, have provided valuable insights into the genetic and regulatory pathways underlying SSD. RNA-Seq allows for the high-throughput quantification of gene expression, making it possible to identify sex-biased genes and pathways that contribute to differential growth between males and females. For example, transcriptome analyses using RNA-Seq have been conducted on species such as Nile tilapia (*Oreochromis niloticus*) [7], loach (*Misgurnus anguillicaudatus*) [8], half-smooth tongue sole (*Cynoglossus semilaevis*) [4], and Chinese mitten crab (*Eriocheir sinensis*) [9], revealing key growth-related genes, including those involved in the growth hormone (GH) and insulin-like growth factor (IGF) pathways. These studies have helped identify differential gene expression patterns that are linked to the sex chromosomes or hormonal regulation, offering a deeper understanding of how SSD manifests at the molecular level.

In addition to conventional RNA-Seq, full-length RNA sequencing technologies, such as PacBio and Oxford Nanopore sequencing, have been developing rapidly in genetics studies, such as whole genome assembly, alternative splicing, and full-length transcripts’ identification [10]. They have been applied to SSD species to capture complete transcript isoforms and resolve complex splicing events, providing a more detailed picture of sex-biased gene regulation. For instance, full-length transcriptome analysis in the three-spine stickleback fish (*Gasterosteus aculeatus*), a species with SSD, has identified sex-specific splice variants and long non-coding RNAs (lncRNAs) that play critical roles in growth and sexual differentiation [11]. In male-biased SSD Hong Kong catfish (*Clarias fuscus*), PacBio full-length sequencing were carried on gonad and testis tissue, which found 5750 testis-biased differentially expressed genes (DEGs) and 6991 ovary-biased DEGs [12]. Similarly, studies in shrimp and crab species, using both RNA-Seq and full-length RNA sequencing, have uncovered novel sex-linked genes and non-coding RNAs involved in growth and metabolism, offering new targets for selective breeding [13]. These advances highlight the importance of integrating RNA-Seq and full-length sequencing to fully capture the complexity of gene expression and regulation in SSD, contributing to more effective strategies for optimizing growth traits in aquaculture. Recent transcriptomic and multi-omics studies in *P. ussuriensis* itself have begun to explore gene expression under environmental stressors, such as acute heat [14] and ammonia exposure [15], revealing pathways related to stress response, liver damage, and metabolic adaptation—underscoring the applicability of these tools to this species and supporting broader efforts to understand its physiology for aquaculture improvement. *Pelteobagrus ussuriensis*, commonly known as the Ussuri catfish, is an economically important freshwater fish species widely cultivated in aquaculture throughout East Asia. This species has garnered significant attention in the aquaculture industry due to its remarkable sexual dimorphism, particularly in growth rates [16]. Males of *P. ussuriensis* exhibit substantially faster growth, reaching approximately three times the size of females under similar rearing conditions. This pronounced sexual dimorphism presents a unique opportunity for aquaculture optimization through sex control techniques. The potential for all-male culture could significantly enhance production efficiency and economic returns for fish farmers. However, despite its commercial importance, the genetic mechanisms underlying sex determination in *P. ussuriensis* remain poorly understood.

Previous studies have suggested an XX/XY sex determination system based on the identification of male-specific genetic markers [16]. Yet, a comprehensive understanding of the sex determination region and potential sex-determining genes has been lacking. This knowledge gap has hindered the development of reliable sex identification methods and sex control strategies for this species. Given the substantial impact that sex-related traits have on aquaculture productivity, elucidating the genomic basis of sex determination in *P. ussuriensis* is crucial for advancing breeding programs and improving aquaculture practices for this valuable catfish species.

## 2. Materials and Methods

### 2.1. Samples Preparation

All *P. ussuriensis* samples were reared in outdoor pond of fish farm of Institute of Aquaculture (30°10′ N, 114°09′ E), Wuhan Agriculture Academy (Wuhan, China). They were reared in pond with 1.5 m depth, and annual temperature was 17 °C. They were fed with pellet feed three times a day and harvested at 13 months past hatch. The body length and body weight info were in Supplementary Table S1. In each sex, there are 3 samples in each large (L) and small (S) group. Before the fish were dissected, they were anesthetized with MS222 (Sigma-Aldrich, St. Louis, MO, USA) in accordance with the Experimental Animal Management Law of China. And then liver and brain tissue from each sample were immersed in liquid nitrogen, then kept at −80 °C. Total RNA was extracted using a RNAiso Plus Reagent Kit (Takara Biotech, Dalian, China) following manufacturer’s instructions. Then genomic DNA within it were removed with TURBO DNase I (Promega, Beijing, China). The quality of RNA was checked with Nanodrop 2100 Bioanalyzer (Agilent Technologies, Palo Alto, CA, USA) and Qubit 3.0 Fluorometer (Life Technologies, Carlsbad, CA, USA). Only these libraries with RIN value > 8 were used for Nanopore sequencing libraries.

### 2.2. Full-Length RNA Sequencing Libraries’ Construction and Sequencing

For each library, 0.5 µg total RNA was used to construct cDNA library following ONT cDNA-PCR Sequencing Kit (SQK-PCS109 + SQK-PBK004, Oxford Nanopore Technologies (ONT), Oxford, UK). In brief, transcriptase was used to perform template-switching to enrich full-length cDNAs and then attach defined PCR adapters to both ends of the first-strand cDNA. Secondly, cDNA PCR was conducted for 14 cycles with LongAmp Tag (NEB, Ipswich, MA, USA). PCR products generated underwent ONT adaptors ligation with T4 DNA ligase (NEB, Ipswich, MA, USA) and Agencourt XP beads were employed for DNA purification. Finally, cDNA libraries were loaded onto FLO-MIN109 flow cells and sequenced on the PromethION platform. All sequence reads have been submitted to the NCBI SRA database under the accession number PRJNA1281719.

### 2.3. Data Processing for Raw Sequencing Reads

Raw sequencing data was saved in fast5 format and then base calling was carried out by Guppy v5.16 [17]. Reads were filtered with a minimum average quality score of 7 and a minimum length of 50 bp by Nanofilt v2.8.0 [18]. Ribosomal RNA (rRNA) was discarded if mapped to rRNA database. Full-length non-chimeric (FLNC) transcripts were identified by searching for two primers at both ends with Pychopper v2.4.0 (https://github.com/epi2me-labs/pychopper, accessed on 24 September 2024). Then, FLNC transcripts were mapped to *P. ussuriensis* reference genome [16] with minimap2 v2.17 [18]. The parameters were set as “-ax splice -uf -k14”. Consensus isoforms were constructed by merging within each cluster using Pinfish v0.1.0 (https://github.com/nanoporetech/pinfish, accessed on 24 September 2024). Then, redundant transcripts were condensed together ignoring 5′ difference with Stringtie v2.1.4 [19].

### 2.4. Annotation File Update

Gff files for each library were merged together and compared with reference annotation file with Gff compare v0.12.1 [20]. In total, there were five types of new transcripts found. Type I is included in intron of reference transcripts. Type j is about multi-exon transcript overlapping at least one exon with annotated transcript. Type o transcripts are located on same strand with other annotated transcripts. Type u transcripts are bona fide transcripts. Type x transcripts are located on anti-sense strand and overlapped with annotated transcripts.

### 2.5. Gene Quantification and Differential Expression Analysis

For gene quantification and differential expression analysis, Nanopore full-length RNA sequencing reads were mapped to the reference transcriptome of *P. ussuriensis* using minimap2 [21] with default parameters, and gene expression levels were quantified in transcripts per million (TPM) using Salmon [22]. Differential expression analysis between fast- and slow-growing groups was conducted using DESeq2 [23]. To identify growth-associated transcriptional differences, analyses were conducted separately within each sex, comparing fast- and slow-growing groups in liver and brain tissues. This stratified design allowed direct characterization of sex-specific growth-related expression patterns. Genes with an adjusted *p*-value (false discovery rate, FDR) < 0.05 and a log_2_ fold change (|log_2_FC|) ≥ 1 were considered significantly differentially expressed. Additionally, differentially expressed transcripts (DETs) were identified using IsoformSwitchAnalyzeR to detect transcript-level changes undetectable at the gene level. Gene Ontology (GO) and Kyoto Encyclopedia of Genes and Genomes (KEGG) pathway enrichment analyses were performed using the clusterProfiler package [24] to identify biological functions and pathways associated with growth regulation.

### 2.6. Alternative Splicing Analysis

Alternative splicing was analyzed using SUPPA2 [25] to quantify splicing events from Nanopore full-length RNA-seq data. Transcript annotations were obtained from Zhu et al. [16] and used to define splicing events. SUPPA2 was employed to calculate Percent Spliced In (PSI) values for each event type, including exon skipping, intron retention, alternative 5′ and 3′ splice sites, and mutually exclusive exons. The PSI values provided a quantitative measure of splicing inclusion levels. Splicing events were considered significantly differential if they showed an absolute change in PSI (|ΔPSI|) ≥ 0.1 between groups and a false discovery rate (FDR) < 0.05. Differentially alternatively spliced genes (DASGs) were defined as genes containing at least one splicing event meeting these criteria.

## 3. Results

### 3.1. Statistics for Full-Length RNA Sequencing

To study the gene expression difference between fast and slow growth and their specificity between male and female in *P. ussuriensis*, three biological repeats were placed in each group for both male and female. Brain and liver tissues were used to study the organ specific influence on growth traits. For all 24 samples, RNA was sequencing on PromethION platform with Nanopore sequencing technology. After filtering low quality reads, there are 6.91 and 4.85 million reads in liver and brain samples on average. And the average length of them is 1054.24 bp and 1475.04 bp in liver and brain, respectively (Supplementary Table S2). The total base for all samples is about 7.19 Gb, which showed no significant difference between liver and brains tissues. To further identify full-length reads with both primers, Pychopper was used and 6.23 million reads were kept on average for each liver sample. While there are 4.11 million reads in each brain sample. Average length for liver and brain samples are 847.58 bp and 1226.94 bp, respectively (Table 1).

### 3.2. New Transcripts and Functional Annotations

**Figure 1:**
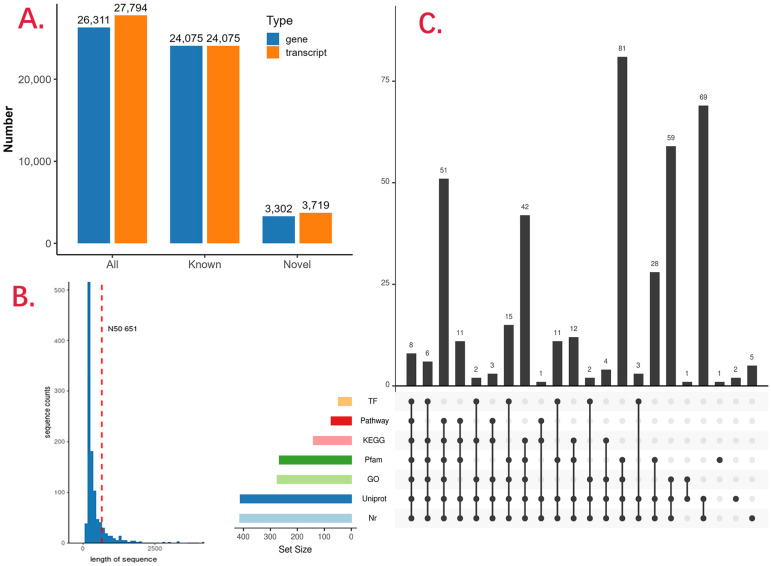
Identification and functional annotation of novel genes and transcripts in *P. ussuriensis*. (**A**) Numbers of newly identified genes and transcripts obtained from the merged transcriptome annotation of all samples. (**B**) Length distribution of coding sequence (CDS) regions of novel transcripts. (**C**) Summary of functional annotation results for novel transcripts based on the NR, KEGG, and KEGG Pathway databases.

We mapped all FLNC transcripts to reference genome and obtained consensus isoforms for each sample (Table 1). Then we merged all samples and obtained a new annotation file for *P. ussuriensis* genome, which contains 3302 new genes and 3719 new transcripts (Figure 1A). Then, the CDS region for all new transcripts were identified, and the N50 for them was 651 bp (Figure 1B). Functional annotations for all 3719 new transcripts showed that 413 (11.11%) obtained results from NR database, 140 (3.76%) obtained results from KEGG database, and 74 (1.99%) were annotated with KEGG Pathway database (Figure 1C, Supplementary Figure S1). Besides these new transcripts, we also extend the gene boundaries for previously existing genes as FLNC transcripts always have precious untranslated region (UTR) information. We found that 3′ UTR of 68 transcripts, 5′ UTR of 29 transcripts, and both 5′ and 3′ UTR of 268 transcripts were extended.

Another updated annotation information was about alternative splicing (AS); we found there are 18,621 AS events in the new annotation file. And for liver tissue, there are 2903.50 AS on average, while there are 7547.88 in brain tissue.

### 3.3. Differential Expression Analysis in Liver and Brain Tissues

**Figure 2:**
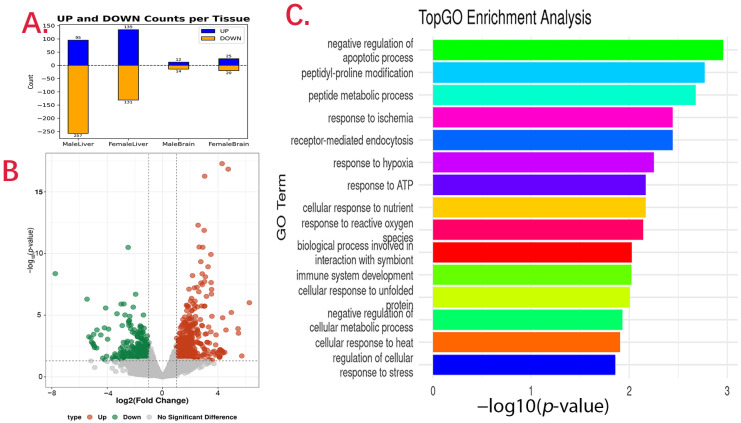
Differentially expressed genes (DEGs) and Gene Ontology (GO) analysis between tissues in *Pelteobagrus ussuriensis*. (**A**) Numbers of upregulated (UP) and downregulated (DOWN) DEGs identified in male liver, female liver, male brain, and female brain. (**B**) Volcano plot showing the distribution of DEGs in female liver tissue. Red dots indicate upregulated genes, green dots indicate downregulated genes, and gray dots represent genes without significant differential expression. (**C**) GO enrichment analysis of DEGs in male brain tissue using TopGO, showing significantly enriched biological process terms.

After mapping all reads to *P. ussuriensis* genome, we were able to do differential expressed genes (DEGs) analysis in male and female samples. The gene expression level was measured by TPM, and its density map showed that all samples were of high quality (Supplementary Figure S2). In liver tissue, we found 332 DEGs between large and small groups in male samples. Among them, 95 were highly expressed in the small group and the rest 237 were highly expressed in the large group (Figure 2A, Supplementary Table S3). Among these top DEGs up-regulated in the large group, there are TMEM135, HSPD1, PA2G4, PGD, and NDRG1. And among these top DEGs up-regulated in the small group, there are VMO1, CPY27A1, SEPT3, SAT1, and LONRF2. KEGG analysis showed that there are eight DEGs that were in PPAR KEGG pathway (ko03320), and they are ANGPTL4, ADIPOR2, LPL, PLIN2, FASN, CYP7A1, CYP27A1, and SLC2A1 (GLUT1). And there are also genes in cholesterol metabolism (ko04979); they are HMGCS1, LDHA, and LPL. Key male-biased DEGs exhibited substantial effect sizes, with log_2_ fold changes ranging from 2.1 to 8.5 (corresponding to 4- to >370-fold higher expression), underscoring their potential role in enhanced metabolic capacity.

While in female samples, 135 DEGs were highly expressed in the small group and another 131 DEGs were highly expressed in the large group (Figure 2A,B, Supplementary Table S4). Among top up-regulated genes in large groups, there are PGBD5, FRMD3, LYZ CYP24A1, and MAP7D1, while in the small group, there are MIOX, PGR, TTR, CXCR4, and CCR9. In these DEGs, several were related to growth regulations, such as IGFBP1, ESR1, and PGR, and many were related to metabolism, such as ELOVL2 and ELOVL6 in lipid metabolism; ALDOB and PDK4 in carbohydrate metabolism; and OAT and BCAT1 in amino acid metabolism. Finally, there are 43 DEGs presented in both male and female comparison (Supplementary Table S5). Among these genes, there are LRAT, DPT, MAP7D1, CYP27A1, PRODH, NR1D1, BCAT1, and RER1.

Following the identification of differentially expressed genes (DEGs), we conducted a comprehensive analysis of differentially expressed transcripts (DETs) to capture isoform-level variations that could further elucidate the complexity of gene regulation in live tissue. The log-transformed transcripts per million (logTPM) values were found to be normally distributed across all samples in both liver and brain tissues (Supplementary Figure S3), indicating consistent and reliable transcript quantification. This normalization ensures comparability between samples and provides a robust foundation for downstream differential expression transcript analyses. In liver tissue of male samples, 8 transcripts in 4 genes (ACOT4, FGFR4 and SAT1) were differentially expressed (Supplementary Table S6), while in female samples, 35 transcripts in 11 genes were also differentially expressed (Supplementary Table S6).

In brain tissue, we only found 26 DEGs between the large and small groups in male samples, 14 of which were highly expressed in the small group while the rest 12 were highly expressed in the large group (Figure 2A, Supplementary Table S7). Gene Ontology enrichment analysis showed that they were enriched in positive regulation of gene expression (GO:0010628), response to external stimulus (GO:0009605), and protein metabolic process (GO:0019538) (Figure 2C). While in female samples, we found 45 DEGs (Supplementary Table S8), 25 of were favored in the small group and the remaining 20 were highly expressed in large group. These genes were enriched in peptide transport (GO:0015833), regulation of response to external stimulus (GO:0032101), and post-translational protein modification (GO:0043687).

### 3.4. Alternative Splicing (AS) Analysis

**Figure 3:**
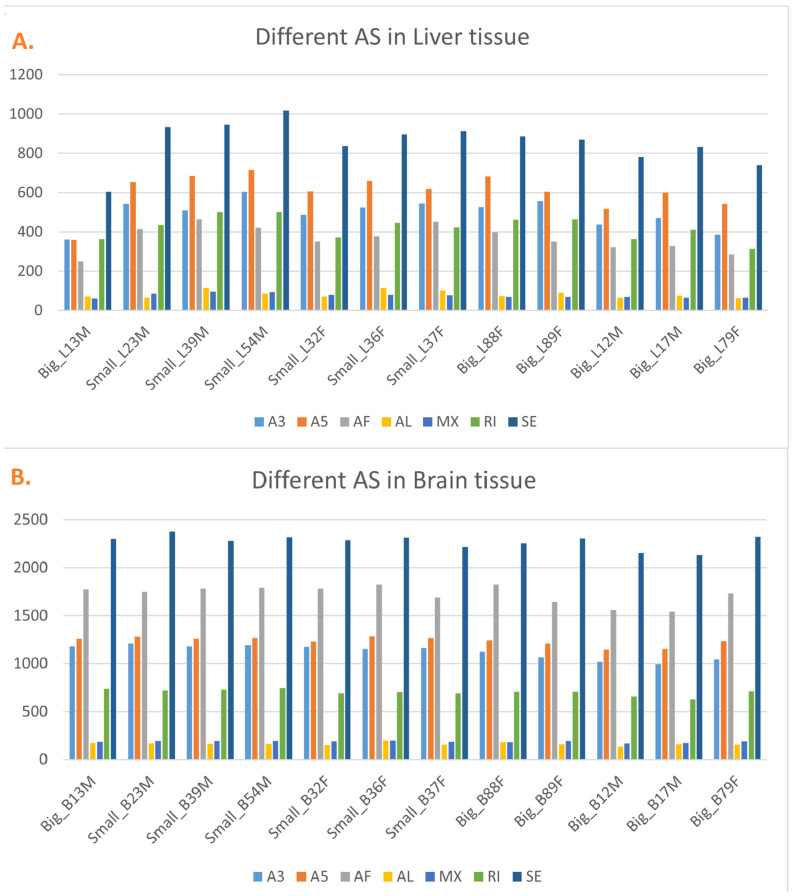
Distribution of alternative splicing (AS) event types in liver and brain tissues of *Pelteobagrus ussuriensis*. (**A**) Numbers of different AS event types identified in liver samples. (**B**) Numbers of different AS event types identified in brain samples. AS events were classified into seven categories, including alternative 3′ splice site (A3SS), alternative 5′ splice site (A5SS), alternative first exon (AF), alternative last exon (AL), mutually exclusive exons (MX), retained intron (RI), and skipping exon (SE).

Following our differential gene expression (DGE) analysis, we explored alternative splicing (AS) events to further elucidate transcriptomic diversity. The use of Nanopore full-length RNA sequencing allowed us to identify and characterize diverse splicing events in brain and liver tissues. On average, 2903 AS events were found in liver tissue while 7412 AS events were found in brain tissue (Figure 3 and Supplementary Table S9). To dive into different types of AS events, skipping exon (SE) is the most abundant one in all samples. While in liver tissue, alternative 5′ splice sites (A5SS) is second to the most frequent type of AS event, which is different from alternative first exon (AF) in brain tissue (Figure 3). The two least frequent AS types are alternative last exon (AL) and mutually exclusive exon (MX) in the two tissues, and their frequency were both lower than 5% of all AS events.

Then, differential AS analyses were carried out in the two tissues. In liver, 60 differentially alternative splicing genes (DASGs) were found between male large and small groups, while 82 DASGs were found in female, by comparison. Many of them were related to cell cycle control (*ATF2*, *FASN*, *IDH1*, and *TPT1*) and cell proliferation (*CAMK4* and *DYRK1A*). As a representative example of growth-associated alternative splicing, *FASN*, a key enzyme in lipid biosynthesis, exhibited a significant exon-skipping event in liver tissue. The inclusion level of this exon was markedly higher in the fast-growing group (ΔPSI = 0.24, FDR < 0.05), while overall *FASN* gene expression showed only a modest change (|log_2_FC| < 0.5). This isoform-specific regulation suggests that alternative splicing, rather than transcriptional regulation, may fine-tune lipid metabolic capacity during rapid growth. Similarly, *SAT1* displayed differential usage of an alternative 5′ splice site in male liver, resulting in distinct transcript isoforms with divergent expression patterns between growth groups, highlighting the contribution of post-transcriptional regulation to growth variation. Then, the same analyses were carried out in brain tissue. A total of 138 and 136 DASGs were found in male and female comparisons, respectively. KEGG analysis for male DASGs found they were enriched in several KEGG pathways like Biosynthesis of amino acids (dre01230), Carbon metabolism (dre01200), and Glycolysis/Gluconeogenesis (dre00010). The female ones were enriched in GO pathways, like structural constituent of ribosome and protein domain specific binding. And for reactome pathways enrichment, they show a more specific pattern of enrichment in genes transcription’s regulation, such as eukaryotic translation initiation (FDR = 4.7 × 10^−2^) and termination (FDR = 8.7 × 10^−3^), Cap-dependent Translation Initiation (FDR = 8.7 × 10^−3^), and Metabolism of proteins (FDR = 2.3 × 10^−2^).

### 3.5. WGCNA for Liver and Brain Tissues

**Figure 4:**
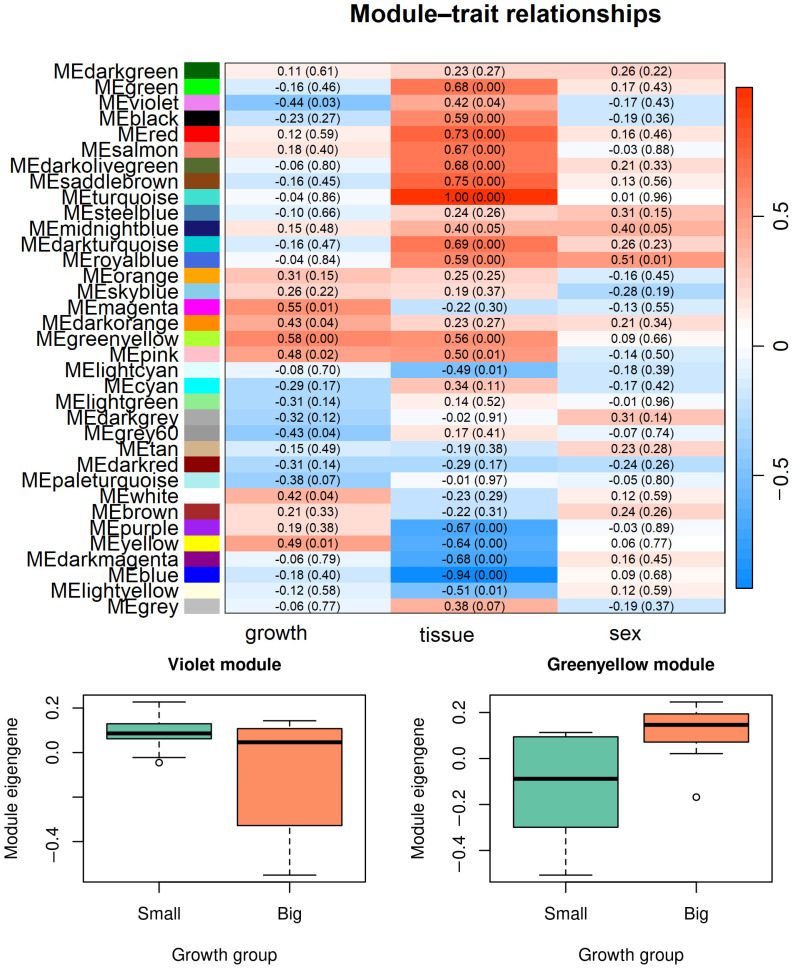
Integrated Weighted Gene Co-expression Network Analysis (WGCNA) results related to growth traits in *Pelteobagrus ussuriensis*. The upper panel shows the heatmap of correlations between module eigengenes and growth-, tissue-, and sex-related traits. Values represent Pearson correlation coefficients with corresponding *p*-values shown in parentheses. The lower panels display eigengene expression patterns of the violet (**left**) and greenyellow (**right**) modules across growth groups (small and big), illustrating growth-associated differences in module activity.

Except for DEGs and DASGs analyses on single genes in previous sections, Weighted Gene Co-expression Network Analysis (WGCNA) was employed to identify gene modules associated with growth rate variations in fish liver and brain tissues. This approach utilizes network theory to detect sets of co-expressed genes across samples, providing insights into the complex gene networks underlying growth phenotypes. Module–trait relationships were assessed by correlating module eigengenes with growth-related traits. Given the exploratory nature of WGCNA, correlations were evaluated at the module level rather than individual gene level. Multiple testing was considered when interpreting module–trait associations, and only modules showing strong correlations and biologically coherent functional enrichment were highlighted. In all 24 tissues, top 85% of expressed genes’ expression value were used to construct the weighted co-expression network. And the best value of power is estimated as 8 (scale-free R^2^ = 0.8, Supplementary Figure S4). Module–trait relationship analysis revealed several modules significantly correlated with growth status, sex, and tissue type (Figure 4). Among these, the violet and greenyellow modules showed strong correlations with growth group, indicating potential involvement in growth regulation. Visualization of module eigengene expression demonstrated that both the violet and greenyellow modules exhibited significantly different eigengene values between the small and big groups, with higher eigengene expression observed in the big group (Figure 4, lower panels). These results suggest that genes within these modules display coordinated expression patterns associated with growth differences.

## 4. Discussions

Growth differences is a crucial trait in economic important animals, significantly impacting productivity and efficiency in various industries such as agriculture and aquaculture [26,27,28]. RNA sequencing has emerged as a powerful tool for identifying key genes responsible for growth variation [29]. Previous studies have utilized RNA-seq to uncover growth-related genes in various species, including livestock and fish [30,31]. In this study, we applied Nanopore full-length RNA sequencing to *P. ussuriensis* to investigate gene expression differences between fast and slow growth samples, as well as sex-specific variations, contributing to the broader understanding of growth regulation mechanisms across species.

Liver and brain are two critical organs in growth regulation, playing distinct but interconnected roles. The liver is central to metabolism, nutrient processing, and the production of growth factors, while the brain is crucial for hormonal control and regulation of growth-related processes [32]. In fish, for instance, the liver is a primary site for insulin-like growth factor (IGF) production, which is essential for somatic growth [33]. The brain, particularly the hypothalamus, regulates the release of growth hormone and other neuroendocrine factors that influence overall growth [34]. Our study’s focus on these two tissues in *P. ussuriensis* provides valuable insights into tissue-specific gene expression patterns related to growth, which may have broader implications for understanding growth regulation across different species.

Nanopore full-length sequencing is gaining more popularity in genomic research due to its ability to capture complete transcript sequences, including novel isoforms and alternative splicing events [35,36]. This technology has proven particularly useful in updating and refining genome annotations [37]. In our study, the application of Nanopore sequencing to *P. ussuriensis* resulted in the identification of 3302 new genes and 3719 new transcripts, significantly enhancing the existing genomic resources. The extension of gene boundaries, including UTR for numerous transcripts, further refines our understanding of gene structure in this species. Moreover, the identification of 18,621 alternative splicing events, with notable differences between liver (2903.50 on average) and brain (7547.88 on average) tissues, highlights the power of full-length sequencing in capturing the complexity of transcriptional landscapes. These findings underscore the value of Nanopore sequencing in improving genome annotations and uncovering previously undetected genomic features, which is crucial for comprehensive studies of gene expression and regulation [38].

The identification of 332 DEGs in male liver tissue and 266 DEGs in female liver tissue between growth groups reveals distinct molecular mechanisms underlying growth regulation in *P. ussuriensis*. In male liver tissue, the upregulation of genes such as *TMEM135*, *HSPD1*, *PA2G4*, *PGD*, and *NDRG1* in the large group suggests their potential roles in promoting growth. *TMEM135* is involved in mitochondrial function and cellular energy metabolism, which are essential for supporting rapid growth [39]. *HSPD1*, a mitochondrial chaperone, facilitates protein folding and stress response, ensuring cellular homeostasis during growth [40]. And *PA2G4* (proliferation-associated 2G4) is known to regulate cell proliferation and apoptosis, processes directly linked to tissue growth and development [41]. Similarly, *PGD* (phosphor gluconate dehydrogenase) and *NDRG1* (N-myc downstream-regulated gene 1) are associated with metabolic regulation and stress adaptation, respectively, both of which are critical for sustaining growth under varying physiological conditions [42]. The enrichment of PPAR signaling pathway genes (*ANGPTL4*, *FASN*, *LPL*) in males aligns with established roles of lipid metabolism in growth regulation, as PPAR pathways modulate energy homeostasis and adipogenesis in vertebrates [34,43]. Similarly, cholesterol metabolism-related DEGs (*HMGCS1*, *LDHA*) suggest coordinated regulation of sterol biosynthesis and glycolysis to meet energy demands during rapid growth, consistent with observations in fish models where cholesterol precursors influence growth hormone signaling [44].

In female liver tissue, genes such as *MIOX*, *PGR*, *TTR*, *CXCR4*, and *CCR9* were up-regulated in the small group. Among them, *MIOX* (myo-inositol oxygenase) has been found involved in carbohydrate metabolism, potentially limiting energy availability for growth [45]. And it is up-regulated in *Arabidopsis thaliana* under conditions in which energy or nutrients are limited [46]. While *PGR* (progesterone receptor) regulates reproductive and metabolic processes, which diverted resources away from somatic growth and caused growth restriction in human population [47]. *TTR* (transthyretin) is involved in thyroid hormone transport and can activate insulin-like growth factor receptor I (IGF-IR) pathways in mice, while higher insulin level always consume more energy and lower body weight [48].

Sex-specific regulatory patterns emerge from our result, with males showing twice as many liver DEGs as females. Key genes such as *IGFBP1* (up-regulated in small-group females) and *ESR1* (differentially expressed across sexes) highlight the interplay between growth hormone signaling and estrogen-mediated pathways in growth dimorphism. These parallel findings in teleosts where estrogen receptors regulate somatic growth by modulating IGF-I expression [27,32]. The identification of 40 DEGs shared between male and female comparisons suggests conserved mechanisms of growth regulation across sexes. These genes are involved in diverse processes, such as retinol metabolism (*LRAT*) [49], extracellular matrix organization (*DPT*), and amino acid metabolism (*BCAT1*), indicating that growth regulation is a multifaceted process involving multiple biological pathways.

Finally, we did an overlap analysis between DEG in current study and gonad transcriptomes in *P. ussuriensis* [50]. For example, *HSPD1*, *PA2G4*, and *NDRG1*, which were significantly up-regulated in fast-growing males in liver tissue, have also been reported as male-biased or testis-enriched genes in gonadal transcriptomes, where they are associated with cellular proliferation, stress response, and metabolic activity. In addition, metabolic regulators such as *FASN*, *LPL*, and *ANGPTL4*, enriched in the PPAR signaling pathway in fast-growing males, overlap with lipid- and steroid-related pathways previously shown to be up-regulated in male gonads. Conversely, several genes showing higher expression in slow-growing females in the present study, including *PGR* and *ESR1*, are consistent with female-biased hormone receptor expression reported in ovarian transcriptomes. Together, these results indicate that sex-biased transcriptional programs identified in gonadal tissues are also reflected in somatic tissues associated with growth, suggesting coordinated regulation of sexual size dimorphism across reproductive and metabolic systems.

Notably, isoform-level analysis uncovered 8 DETs in male liver (e.g., *ACOT4*, *SAT1*) and 35 in female liver that were undetectable at the gene level, underscoring the value of full-length sequencing for resolving transcriptional complexity. This mirrors findings in goldfish, where breed-specific alternative splicing generated phenotypic diversity despite similar gene expression profiles [36]. The detection of *SAT1* splice variants in small-growth males suggests post-transcriptional regulation of polyamine metabolism—a known growth modulator in fish [51].

In brain tissue, the limited DEGs (26 in males, 45 in females) contrasted sharply with hepatic profiles, reflecting tissue-specific regulatory strategies. The enrichment of response to external stimulus GO terms in male brain DEGs implies neural modulation of growth through environmental sensing mechanisms, as documented in salmonids where photoperiod sensing regulates growth hormone axis [52]. Conversely, female brain DEGs associated with peptide transport (*TTR*, *CXCR4*) may coordinate nutrient allocation to reproductive tissues, consistent with life-history trade-offs between growth and reproduction in iteroparous species.

The analysis of alternative splicing (AS) events in liver and brain tissues revealed significant transcriptomic diversity associated with growth differences in *P. ussuriensis*. In liver tissue, skipping exon (SE) was the most frequent AS event, followed by alternative 5′ splice sites (A5SS), while in brain tissue, alternative first exon (AF) ranked second. These tissue-specific patterns suggest that AS mechanisms are tailored to the functional demands of each tissue, with A5SS potentially regulating metabolic processes in the liver and AF contributing to transcriptional complexity in the brain. The low frequency of alternative last exon (AL) and mutually exclusive exon (MX) events (<5%) highlights their specialized roles in fine-tuning gene expression, possibly influencing growth-related processes such as protein localization and interaction networks [53].

Differential AS analysis identified key genes involved in cell cycle control (*ATF2*, *FASN*, *IDH1*, *TPT1*) and proliferation (*CAMK4*, *DYRK1A*), underscoring the role of AS in modulating growth. In brain tissue, DASGs were enriched in pathways like amino acid biosynthesis and glycolysis, linking AS to energy metabolism and nutrient utilization critical for growth. The use of Nanopore full-length RNA sequencing was instrumental in capturing rare and complex AS events, demonstrating its advantage over short-read technologies in uncovering transcriptomic diversity. These findings highlight the importance of AS in growth regulation and provide a foundation for further functional studies on specific splicing isoforms and their roles in growth modulation, which has been reported many times in different plants [54].

## 5. Conclusions

Our study leveraging Nanopore full-length RNA sequencing provides novel insights into the transcriptional landscape underlying growth differences in male and female *Pelteobagrus ussuriensis*. By analyzing liver and brain tissues, we identified substantial gene expression and alternative splicing variations associated with growth regulation, highlighting tissue-specific and sex-dependent molecular mechanisms. The identification of key DEGs in difference sex, such as those involved in metabolic pathways, hormonal regulation, and energy homeostasis, underscores the complex interplay of genetic and physiological factors in growth modulation. Furthermore, the discovery of numerous alternative splicing events, particularly in the brain, reveals an additional layer of transcriptional signature relevant to growth phenotypes. The sex-specific differences in gene expression and isoform diversity further emphasize the role of differential regulatory strategies in mediating growth variation. Our findings not only enhance the genomic resources available for *P. ussuriensis* but also contribute to the broader understanding of growth regulation mechanisms across species. To further verify the biological function of these genes, more experiments such as qPCR and protein-level assays were needed in the future. The application of long-read sequencing technologies continues to refine our knowledge of transcriptome complexity, paving the way for future functional studies to elucidate the precise roles of identified genes and isoforms in growth regulation.

## Figures and Tables

**Table 1:** Summary statistics of sequencing reads and mapping efficiency for each sample.

Sample	Num_Seqs	Sum_Len	Avg_Len	Max_Len	Mapped Reads	MAP Ratio (%)
Big_L13M	6,243,260	5,500,360,347	881	11,281	6,210,018	99.47
Small_L23M	6,488,756	5,538,717,284	853.6	11,270	6,463,999	99.62
Small_L39M	5,710,442	5,379,406,470	942	11,335	5,688,350	99.61
Small_L54M	5,977,635	5,617,154,520	939.7	13,425	5,954,588	99.61
Small_L32F	5,976,402	5,567,501,746	931.6	11,304	5,956,821	99.67
Small_L36F	6,808,252	5,842,292,645	858.1	13,484	6,777,775	99.55
Small_L37F	6,016,857	5,607,440,165	932	11,931	5,992,895	99.6
Big_L88F	6,994,264	5,655,388,815	808.6	11,271	6,954,942	99.44
Big_L89F	7,401,888	5,044,574,171	681.5	11,182	7,350,740	99.31
Big_L12M	5,030,934	4,338,002,348	862.3	10,987	4,208,596	99.32
Big_L17M	6,339,910	4,559,610,835	719.2	10,397	4,253,776	99.16
Big_L79F	5,842,102	4,448,342,813	761.4	13,684	3,661,122	99.1
Big_B13M	4,237,574	6,164,541,459	1454.70	12,036	3,993,242	99.24
Small_B23M	4,289,950	5,558,312,802	1295.70	13,005	4,114,231	99.15
Small_B39M	3,694,396	5,036,813,496	1363.40	12,617	3,921,816	99.19
Small_B54M	4,023,825	5,241,741,375	1302.70	15,128	4,364,553	98.95
Small_B32F	4,149,709	5,141,111,928	1238.90	11,750	3,643,387	99.07
Small_B36F	3,953,684	5,621,352,473	1421.80	14,063	4,447,202	98.78
Small_B37F	4,410,915	5,047,558,890	1144.30	13,950	5,010,642	99.6
Big_B88F	3,677,623	4,623,990,492	1257.30	14,204	6,296,881	99.32
Big_B89F	4,502,334	5,116,789,715	1136.50	14,332	4,848,062	98.72
Big_B12M	4,910,946	4,455,821,893	907.3	10,076	4,043,312	98.96
Big_B17M	4,085,855	3,918,535,291	959	10,490	5,806,134	99.38
Big_B79F	3,495,440	4,340,322,071	1241.70	13,485	3,446,082	98.59

## Data Availability

The raw data supporting the conclusions of this article will be made available by the authors on request.

## Supplementary Materials

The following supporting information can be downloaded at: https://www.mdpi.com/article/10.3390/ani16030439/s1, Figure S1: GO and KEGG functional annotations for all new transcript. (A) GO annotaions for biological process, molecular function and cellular component. (B) KEGG annotations in different pathways. Figure S2: TPM density plot for all 24 samples. Figure S3: Box plot of TPM values for all 12 samples in liver tissue. Figure S4: Determination of the soft-thresholding power for WGCNA. Table S1: The body length and body weight info for all 24 samples used in this study. Table S2: Summary statistics of all 24 samples. Shown are the total number of reads (num_seqs), cumulative read length (sum_len), average read length (avg_len), and maximum read length (max_len) for each sample. Table S3: All 332 DEGs between large and small groups in male liver samples. Table S4: All 266 DEGs between large and small groups in female liver samples. Table S5: All 43 DEGs between large and small groups in male brain samples. Table S6: All 15 genes with DETs in male and female liver samples. Table S7: All 26 DEGs between the large and small groups in male brain samples. Table S8: All 45 DEGs between the large and small groups in female brain samples. Table S9: Summary statistics for different AS events in all 24 samples.
