# Supplementary figures and images for: Differential Expression and Alternative Splicing Pattern in Female and Male Groups Pelteobagrus ussuriensis with Different Growth Rate
Source: Animals (Basel). 2026 Jan 30;16(3):439. doi: 10.3390/ani16030439 (PMC12896394; doi:10.3390/ani16030439)

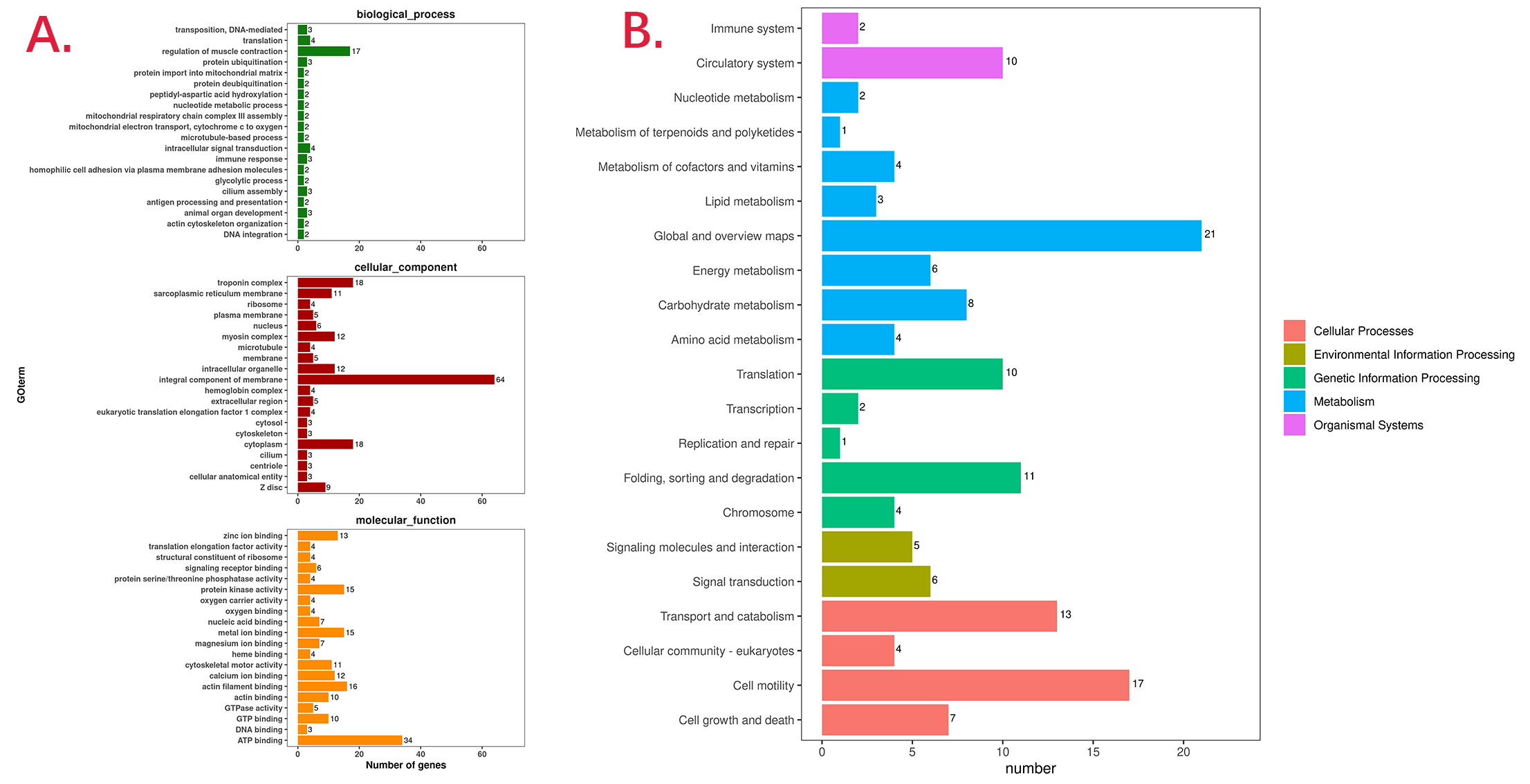

Supplement: Supplementary file 1 [file animals-16-00439-s001.zip › animals-4092070-supplementary figures/Supp Figure S1.tif]

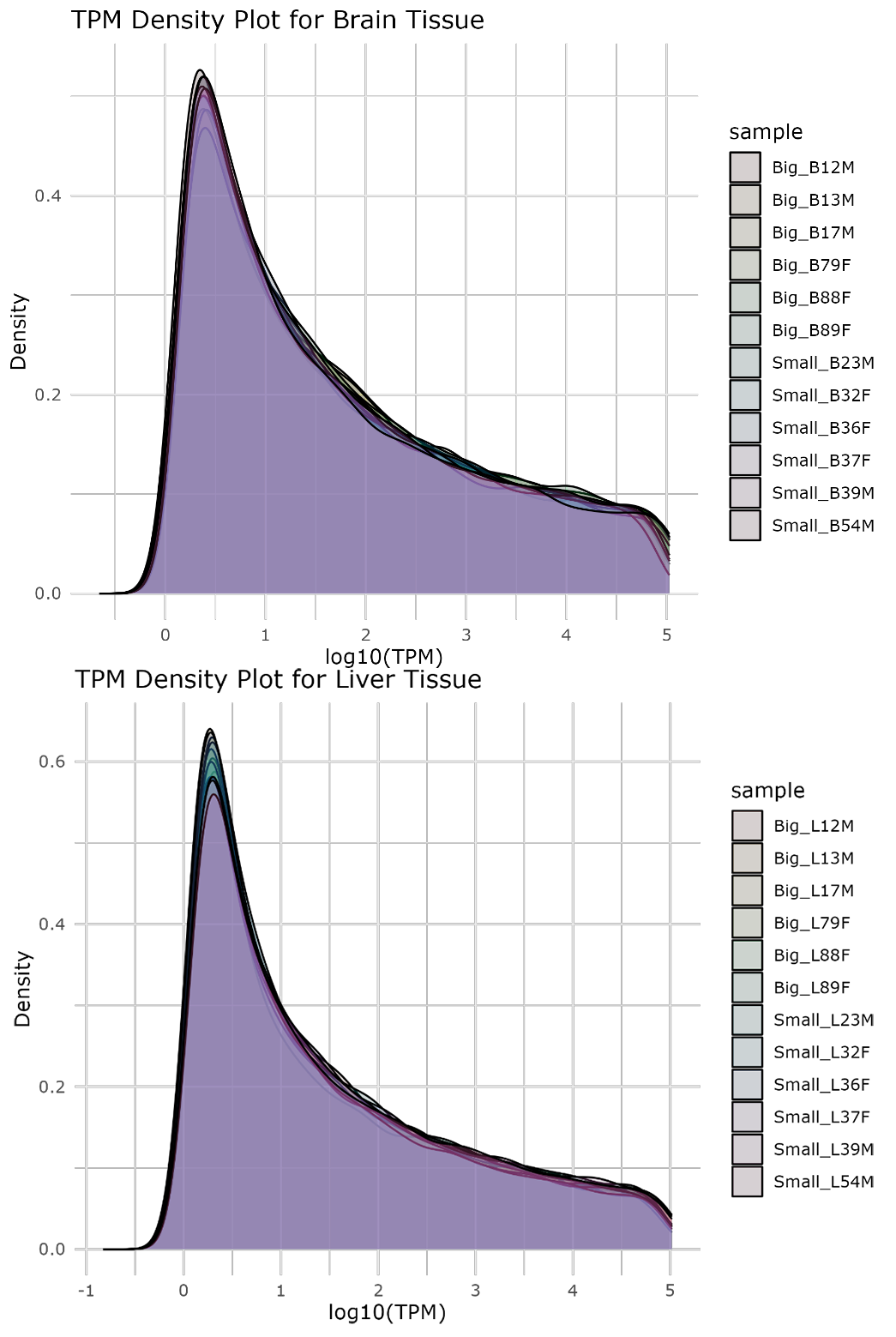

Supplement: Supplementary file 1 [file animals-16-00439-s001.zip › animals-4092070-supplementary figures/Supp Figure S2.tif]

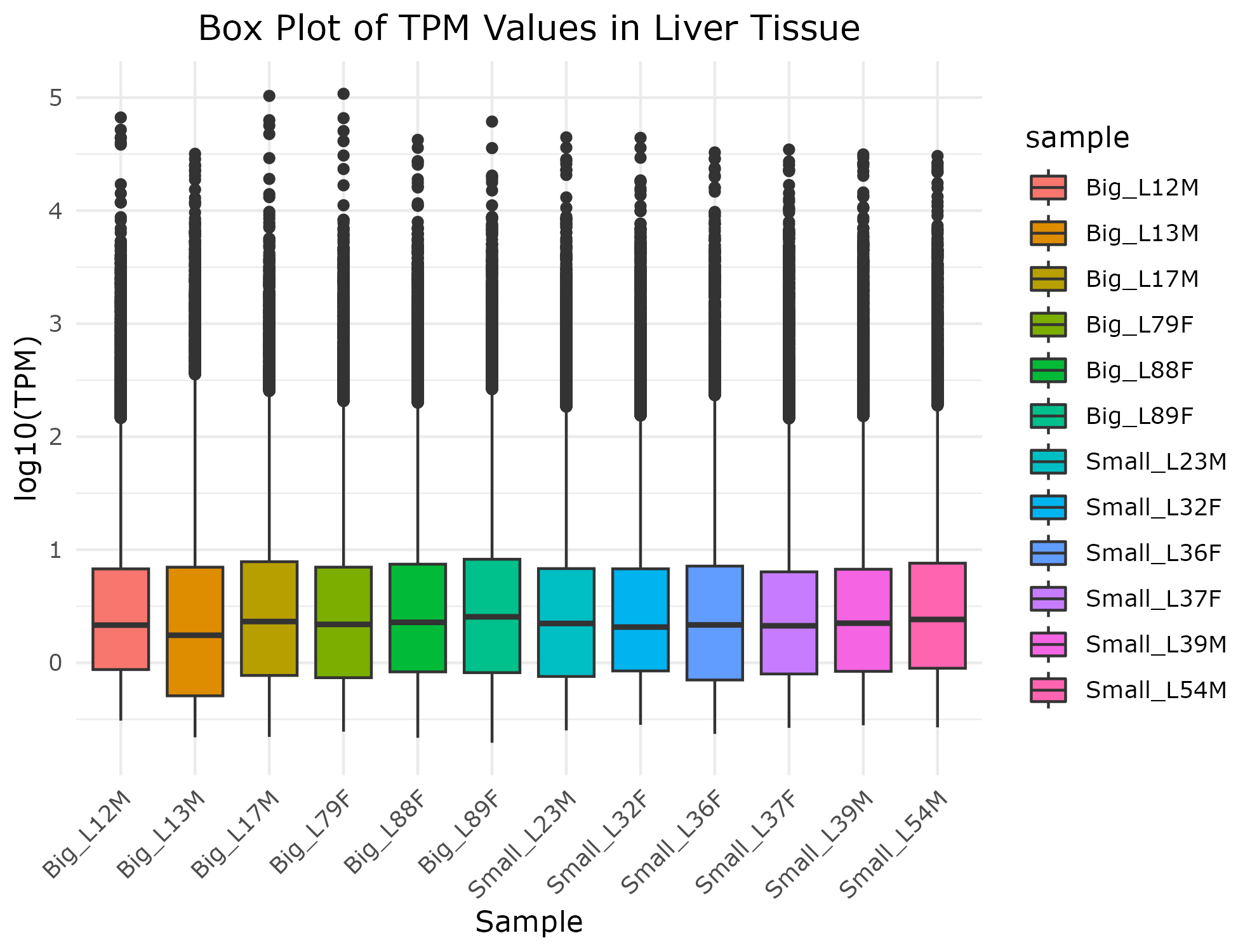

Supplement: Supplementary file 1 [file animals-16-00439-s001.zip › animals-4092070-supplementary figures/Supp Figure S3.tif]

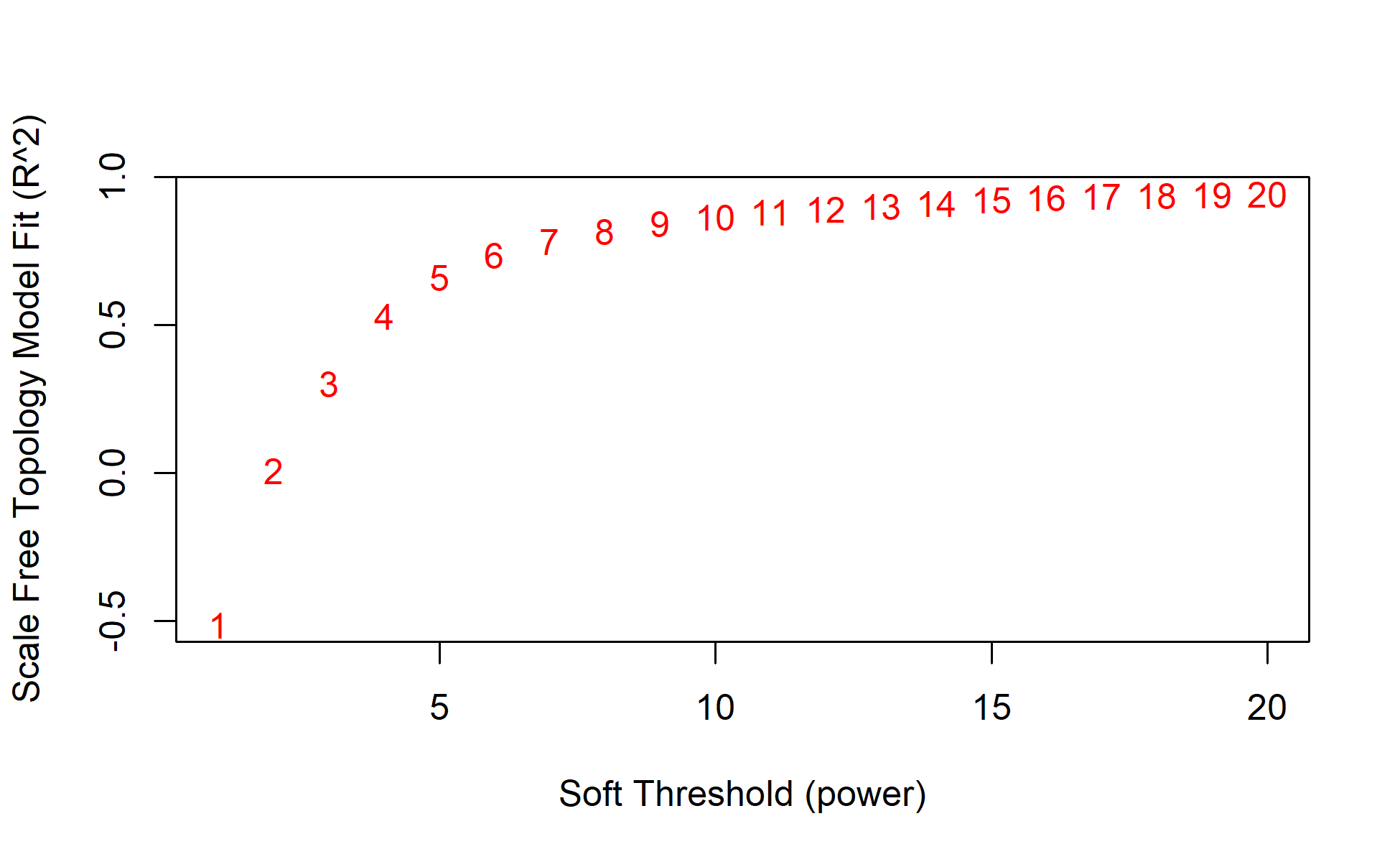

Supplement: Supplementary file 1 [file animals-16-00439-s001.zip › animals-4092070-supplementary figures/Supp Figure S4.tiff]
